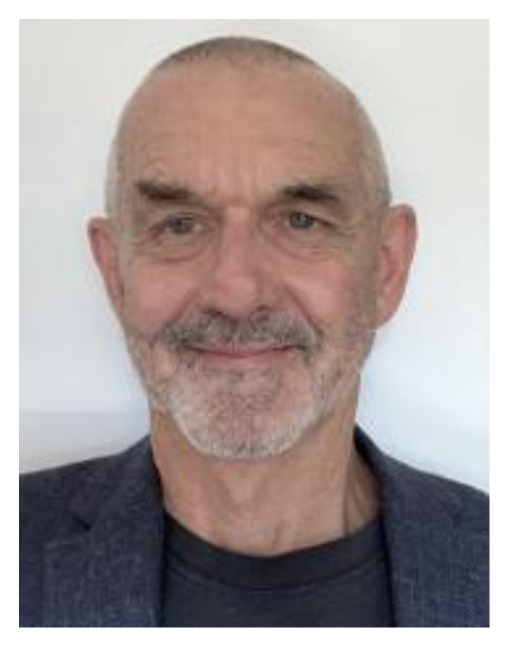# Reflecting on 2025 and Looking Ahead

**DOI:** 10.17159/2078-516X/2026/v38i1a25343

**Published:** 2026-01-15

**Authors:** Mike Lambert

**Affiliations:** Editor-in-chief

As we enter 2026, the South African Journal of Sports Medicine (SAJSM) pauses to reflect on the past year’s achievements and to focus on the opportunities ahead. This annual reflection recognises the collaborative efforts of our authors, reviewers, readers, and editorial team, reaffirming our dedication to ethical, high-quality scholarship in sports medicine and related fields. In a growingly complex global publishing landscape, our mission remains clear: to be a trusted platform for research that advances knowledge and practice, especially within South Africa.

The past year demonstrated the journal’s increasing reach and significance. We recorded 7,700 downloads from our website, a sign of the ongoing interest in the work we publish and its usefulness to a wide range of clinicians, researchers, educators, and students. We received 66 submissions and published 32 papers. Our acceptance rate of just over 50% reflects our commitment to maintaining high standards. Papers are most often rejected due to poor research design or a failure to align with the journal’s objectives and scope—a reminder to prospective authors of the importance of methodological rigour and relevance to our focus areas.

A highlight of the past year was the diversity of institutions represented in our published manuscripts. We are proud to have featured contributions from all major universities in South Africa, including Stellenbosch University, the University of Cape Town, the University of Pretoria, the University of the Free State, the University of Johannesburg, Tshwane University of Technology, UNISA, Durban University of Technology, the University of KwaZulu-Natal, Cape Peninsula University of Technology, the University of the Western Cape, North-West University, the University of Fort Hare, Sefako Makgatho Health Sciences University, and the University of the Witwatersrand. This broad representation confirms SAJSM’s role as a genuinely national platform, bringing together the country’s academic and clinical communities in a unified pursuit of excellence in sports medicine.

Our reach goes beyond South Africa. We published work from international institutions such as Leeds Beckett University, Amsterdam UMC, and the University of Namibia, as well as contributions from Burkina Faso. These global connections enhance our content, and help SAJSM be part of a worldwide conversation while staying focused on regional issues.

On the technical and operational front, we made notable progress. We improved our website metadata by including identification of institutions linked to a research organisation registry (ROR), XML downloadable files, references, and keywords. These enhance online discoverability and ensure compliance with modern publishing standards. Production efficiency also improved, with published papers now appearing in the PubMed database within 4 weeks—a critical step in guaranteeing timely visibility for authors and integration into the global literature. Additionally, we expanded our online presence by launching a LinkedIn profile alongside our existing X platform, enabling us to engage diverse audiences and share updates on new publications and discussions.

Peer review remains the foundation of our credibility, and we sincerely thank our reviewers for their efforts over the past year. Most provided high-quality reviews, offering thoughtful feedback that improves the work we publish. However, as is typical in academic publishing, finding willing and suitable reviewers remains challenging. We are committed to exploring ways to support and recognise this vital work, knowing that without it, maintaining our standards would be impossible.

Operationally, we navigated a challenging phase with payments for page charges, but we are pleased to report that the payment portal on the South African Sports Medicine Association (SASMA) website has been updated. While page charges are necessary to sustain the journal, we aim to make the process as smooth as possible for authors.

The responsibility for maintaining high standards rests with our small but dedicated editorial team. Special thanks go to Dr Kathryn van Boom (journal manager), Ame-Leigh Daniels (copyeditor), and Robyn Lunn-Collier (typesetter). They are, frankly, overworked and underpaid, yet their enthusiasm and commitment shine through in every publication. Their efforts form the backbone of SAJSM. This dedication is especially vital in a publishing environment increasingly affected by money-driven predatory journals and AI-generated, low-integrity content. These trends present a real challenge to journals like SAJSM, which must navigate ethical, high-quality publishing amidst noise and opportunism.

We are deeply grateful to the Academy of Science of South Africa (ASSAf), which manages our online platform and provides ongoing training for editorial staff across South African journals. Their efforts help us stay current with the rapidly changing field of publishing and ensure we follow best practices in open-access scholarship.

As a measure of our standing, we note that SAJSM is ranked in the Q3 by Scimago in both the *Orthopaedics and Sports Medicine* and the *Physical Therapy, Sports Therapy, and Rehabilitation* categories, with an h-index of 8. While metrics are only part of the story, they reflect our growing visibility and encourage us to keep pushing forward.

Looking to 2026, we are filled with anticipation. We already have excellent papers ready for publication, and we expect another year of impactful research and meaningful dialogue. To our authors, reviewers, readers, and the broader SASMA community: thank you for sustaining SAJSM. Together, we will continue to build a journal that stands for integrity, relevance, and excellence in sports medicine.

**Figure:**